# Carer burden in rare inherited diseases: a literature review and conceptual model

**DOI:** 10.1186/s13023-022-02561-w

**Published:** 2022-12-09

**Authors:** Kerry Sandilands, Angela Williams, Angela J. Rylands

**Affiliations:** Kyowa Kirin International, Marlow, UK

**Keywords:** Carer, Burden, Rare disease, Inherited, Quality of life, Literature review

## Abstract

**Background:**

Carers of people living with rare diseases report heavy burden and a plethora of unmet needs. A previous parental supportive care needs framework has described the needs of parents of children living with rare diseases, but it is not specific to rare *inherited* diseases (RIDs) and does not include non-parental carers. We conducted a targeted literature review to: (1) ascertain the burden/supportive care needs of informal carers of people living with RIDs, (2) understand the burden/supportive care needs unique to these carers, and (3) develop a conceptual model based on the findings.

**Methods:**

A targeted literature review searching Embase and Medline between 2000 and 2020 was conducted to identify journal articles describing the burden/supportive care needs of all types of informal carers of people living with RIDs. Thematic analysis was conducted on the articles to develop a conceptual model.

**Results:**

After screening and quality appraisal, 31 journal articles were analysed, representing 70 RIDs (including bleeding, bone, central nervous system, multisystem and inherited metabolic disorders). Most articles (74%) focused on parent carer samples. The conceptual model has three overarching domains, encompassing 13 themes: (1) Living with Rare Inherited Disease (Being a Carrier of Rare Disease, Carer Perceptions, Disease Severity); (2) Carer Needs/Burden (Social/Community, Well-being, Information, Practical); and (3) Carer Coping Strategies (Acceptance, Support Systems, Gratitude and Hope, Faith, Quest for Knowledge, Establish a Routine). Our conceptual model uniquely describes carers’ transmission guilt, clinically relevant depression and anxiety, worry about future family members living with the RID, and challenging decisions about having more children. Carers often implemented psychological, structural, practical, and social coping strategies to manage their burdens.

**Conclusions:**

The identified burdens underscore the need for the provision of information and social support to these carers. Future research should focus on the (1) potential mediators/moderators of carers’ burden, (2) needs of carers within the wider family including siblings and grandparents, (3) needs of carers of adults living with RIDs, including spouses and children, and (4) biopsychosocial effect on carers living with a RID themselves. Our conceptual model offers a potential tool for healthcare professionals to utilise during the provision of support to carers.

## Background

Rare diseases greatly disrupt the lives of those living with these conditions [1, 2], and those of their families/carers [3]. Informal carers (i.e., those who assist a person living with a disability or chronic disorder with their activities of daily living in an unpaid role [4]) aid people living with rare diseases in many ways, including through medication administration [5], mobility support [6], and attendance at healthcare appointments [6, 7]. Consequently, informal carers of people living with rare diseases often report extensive hours spent each week providing care [8], and negative effects on their quality of life (QoL; e.g., stigma [9] and disrupted mental health [10]) in the face of limited external psychological, social, emotional, and financial support [11]. Two previous literature reviews [12, 13] explored how caring for a child living with a rare disease affects parent carers’ lives; Boettcher et al. specifically focused on carer QoL/its predictors [13], whereas Pelentsov et al. [12] built upon a pre-existing supportive care needs framework (SCNF) to develop the parental SCNF, a detailed summary of the psychological, emotional, practical, social, informational, and physical needs of these parent carers.

Of the 6000 + rare diseases, 71.9% are genetic and of those, 79.9% are inherited. Thus, approximately half (57.5%) of the genetic rare diseases are inherited [14]. However, the specific implications of caring for someone living with a rare *inherited* disease (RID), where the parent carer may be a carrier of the disease and also be living with the same condition, has not been clearly described by prior literature reviews. Moreover, parent carers have been the predominant focus of these literature reviews, limiting the understanding of how different informal carers (e.g. spouse, sibling, partner) are affected by their caring role.

To address these gaps, we conducted a targeted literature review aiming to: (1) identify key literature to ascertain the supportive care needs and burden of informal carers of people living with RIDs, (2) identify needs of carers that are unique to RIDs, and (3) develop a conceptual model based on these findings. We drew upon Pelentsov et al. [12] and their parental SCNF as a basis for the search strategy/key word search and data extraction, and conducted thematic analysis [15] on the included articles to develop a conceptual model.

## Methods

The targeted literature review was conducted in two phases: 1) identification of literature, quality appraisal and data extraction; and 2) thematic analysis and the development of a conceptual model.

### Phase 1: Targeted literature review

#### Data sources and search strategy

AN electronic keyword search of titles and abstracts was undertaken by one researcher using the platform ProQuest to search the Medline and Embase databases between 1 January 2000 and 31 December 2020 (see Table 1 for full search terms). The same researcher screened the 169 retrieved records, which were only retained if they described self-reported qualitative/quantitative impacts/experiences/burdens/supportive care needs of informal or unpaid carers of people living with RIDs in the following domains: practical, physical, informational, emotional, psychological, or social. Another criterion for review was the article had to concern carers of people with a condition that is either listed as a RID in the National Organization for Rare Disorders database [16] or identified as a RID in the article’s title/abstract. Only articles published within the search period were included. Case studies, conference abstracts, editorial articles, and non-English publications were excluded. The full text of relevant articles were obtained for further screening against the inclusion criteria. Following screening, the reference lists of retained full-text journal articles describing primary research were reviewed for additional relevant articles meeting the inclusion criteria, using a backward snowballing approach [12].

**Table 1:** Keywords used to search electronic databases

(TI,AB(Rare condition[*1]) OR TI,AB(Rare disease[*1]) OR TI,AB(Orphan disease[*1])) AND (TI,AB(Inherited disease[*1]) OR TI,AB(Genetic disease[*1]) OR TI,AB(Hereditary disease[*1])) AND (TI,AB(Caregiver[*1]) OR TI,AB(Carer[*1])) AND (TI,AB(Experience[*1]) OR TI,AB(Impact[*1]) OR TI,AB(Need[*1]) OR TI,AB(Burden[*1]) OR TI,AB(Support[*1]))	

#### Data review and quality control

The quality of all journal articles identified after screening and snowballing was assessed. Articles describing a quantitative methodology were assessed using a record-grading scale informed by the NICE Quality appraisal checklist for quantitative studies [17]. For articles describing a qualitative, mixed-method, case–control, and literature review methodology the CASP- Qualitative Studies Checklist [18], Mixed Methods Appraisal tool (MMAT) Version 2018 [19], CASP—Case Control Study Checklist [20], and the CASP-Systematic Review Checklist [21], respectively, were used.

#### Data extraction

After quality appraisal, the following data were extracted from the remaining journal articles into an Excel file: (1) study design; (2) year of publication; (3) RID of focus; (4) carer relationship to the person living with the condition; (5) number of carers included in sample; (6) age and gender of carers and (7) their care recipient; (8) location and region of study; and (9) summary data outlining carers’ burden/supportive care needs.

### Phase 2: Development of data-driven conceptual model

#### Data analysis

All full-text articles were uploaded into NVivo® software [22] (v12), and thematic analysis [15] was conducted, analysing key statements from the results and discussion sections of the articles (KS and AR). Researchers first familiarised themselves with the data, reading the articles and extracting relevant information, as per Sect. “Data extraction”. Key statements were then coded (excluding direct participant quotes) by one researcher (KS) implementing an inductive bottom-up approach. Codes were reviewed by a second researcher (AR) to ensure consistency and to minimise bias. Subsequently, codes were arranged into similar groupings by both researchers and were refined to identify initial themes. Codes that were grouped in a similar manner by the two researchers remained grouped, and codes that were grouped differently were discussed together until mutual agreement was reached.

#### Development of data-driven conceptual model

Following data analysis, AR and KS met to discuss the structure and visual representation of the conceptual model based on the results of thematic analysis. The initial model was then reviewed by two separate researchers, who suggested changes to theme names and code groupings. This included the grouping of similar codes under one subtheme of Support Systems, developing a new theme name of Carer Perceptions, alongside re-organising codes within Well-being.

## Results

### Screening and quality appraisal process

**Fig. 1:**
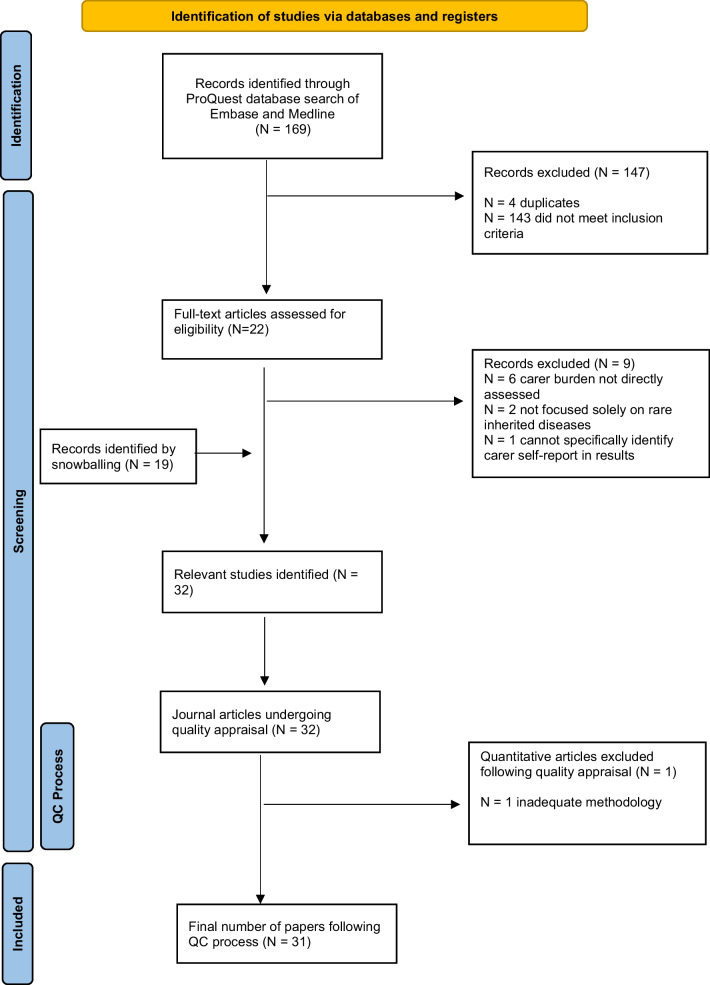
PRISMA flowchart

A PRISMA flowchart detailing the article selection process is illustrated in Fig. 1. In summary, 169 records were retrieved from the initial Embase and Medline database search, and 22 journal articles remained after screening of titles and abstracts. After full-text review, 13 remained. The reference lists of the 10 primary research journal articles (excluding three literature reviews) were reviewed via backward snowballing, resulting in 19 additional eligible journal articles being retained. Following quality assessment of the 32 journal articles, one quantitative survey study was removed due to methodological inadequacies, resulting in 31 journal articles being included in thematic analysis.

A moderate risk of bias (e.g., small sample sizes) was identified in 11 of the 17 included quantitative studies. Of the remaining study designs included in this review (qualitative, mixed-methods, case–control and literature review), some methodological limitations were identified. For example, biased sampling strategies were employed in some qualitative papers, however, most articles described a robust analysis process, with more than one researcher involved. For literature reviews only one of the four papers described a quality appraisal process and for case–control and mixed-methods studies there was limited use of validated questionnaires.

### 3.2 Publication characteristics and demographic data of carers

The final journal articles included are summarised in Table 2 according to study characteristics, including study participants’ demographic data. In summary, the 31 articles (four literature reviews, eight qualitative, two mixed-methods, and 17 quantitative journal articles) covered 70 RIDs, including bleeding disorders (e.g., haemophilia A and B, sickle cell anaemia), dermatological disorders (e.g., epidermolysis bullosa), bone disorders (e.g., osteogenesis imperfecta), central nervous system disorders (e.g., spina bifida), inherited metabolic diseases (e.g., maple syrup urine disease, tyrosinemia type I), and multisystem disorders (e.g., Zellweger spectrum disorder, mucopolysaccharidosis, Von Hippel–Lindau disease). Articles were published between 2005 and 2020 and focused on carers living in Europe, North America, South America, Asia, and Oceania. Twenty-three articles (74%) focused solely on parent carer samples, six articles (20%) focused on parents and other family members, and two articles (6%) did not report the specific carer population of focus. The average number of carers within quantitative studies was 117 (range 11–561).

**Table 2:** Articles included in the review

Title	Study design	Publication year	Disease (s) coded during thematic analysis	Type of carers	Relationship to patient (%)	Number of carers	Age	Countries	Concepts
Health state preference scores of children with spina bifida and their caregivers	Quantitative cross-sectional	2005	Spina bifida	Parents Grandparents	Mother (96%) Father/Grandmother (4%)	98	Mean (range): 37.7 (24–70)	NR	Well-being
Caring for the carers: quality of life in Huntington’s disease	Literature review	2005	Huntington’s disease	N/A	N/A	N/A	N/A	N/A	Practical Support Systems Well-being
The main problems of parents of a child with epidermolysis bullosa	Qualitative	2008	DDEB EBS EBS-DM EBS-K EBS-WC JEB-nH RDEB RDEB-mut	Parents	Mother (64.7%) Father (35.3%)	17	NR	Netherlands	Information Practical Social/Community Well-being
Quality of life in patients with epidermolysis bullosa	Quantitative cross-sectional	2009	DDEB, generalised DDEB, other subtypes EBS-DM EBS, localised EBS, other subtypes JEB-nH, generalised KS RDEB, generalised other RDEB, other subtypes RDEB, severe generalised	N/R	N/R	125	N/R	N/R	Social/Community Well-being
Family burden in epidermolysis bullosa is high independent of disease type/subtype	Quantitative cross-sectional	2010	DDEB, generalised EBS-DM EBS, localised EBS, other subtypes JEB n–H, generalised RDEB, generalised other RDEB, other subtypes RDEB, severe generalised	Parents	Mother (82.1%) Father (17.9%)	28	Mean: 34.4	Italy	Disease Severity Faith Information Practical Social/Community, Support Systems Well-being
Factors affecting quality of life in epidermolysis bullosa	Literature review	2010	DDEB EBS JEB RDEB	Parents	NR	NR	NR	NR	Information Practical Social/Community Well-being
Caregiver’s burden and quality of life in mitochondrial disease	Quantitative cross-sectional	2010	Complex I defect	Parents	Mother (100%)	33	Mean (range): 37.72 (29–55)	Korea	Information Practical Well-being
The burden and quality of life of caregivers of sickle cell anemia patients taking hydroxyurea versus those not taking hydroxyurea	Quantitativecross-sectional	2012	Sickle cell anaemia	Parents	Mothers (81.1%) Fathers (18.9%)	37	Mean (range): 42.95 (18–69)	Brazil	Practical Well-being
Assessment of quality of life of parents of children with osteogenesis imperfecta	Quantitative cross-sectional	2012	Osteogenesis imperfecta types I, III, and IV	Parents	Mother (NR) Father (NR)	25	Mothers, mean: 34.3 Fathers, mean: 38.1	Poland	Disease Severity Practical Social/Community Support Systems
The experience of being a female carrier of haemophilia and the mother of a haemophilic child	Qualitative	2013	Haemophilia A Haemophilia B	Parents	Mothers (100%)	13	Mean (range): 44 (28–83)	Sweden	Being a Carrier of Rare Disease Carer Perceptions Information Practical Social/Community Support Systems Quest for Knowledge Well-being
Understanding the experience of caring for children with haemophilia: cross-sectional study of caregivers in the United States	Quantitative cross-sectional	2014	Haemophilia A Haemophilia B	Parents Other relatives	Mothers (88.39%) Fathers (10.3%) Grandparents or siblings (1.3%)	310	Age Category: mean (%) 18–34: 76 (24.5%) 35–44: 148 (47.7) 45–54: 82(26.5) 55–64: 4(1.3)	US	Disease Severity Practical Well-being
Shaping and managing the course of a child’s disease: parental experiences with osteogenesis imperfecta	Qualitative	2014	Osteogenesis imperfecta	Parents	Mothers (70.83%) Fathers (29.17%)	48	NR	Canada, US, Latin America, Europe	Acceptance Being a Carrier of Rare Disease Carer Perceptions Information Practical Social/Community Well-being
Parents of children with haemophilia at an early age: assessment of perceived stress and family functioning	Quantitative cross-sectional	2014	Haemophilia A Haemophilia B	Parents	Mothers (53%) Fathers (47%)	49	Mean: 38.86	Spain	Practical Well-being
Haemophilia Experiences, Results and Opportunities (HERO) Study: influence of haemophilia on interpersonal relationships as reported by adults with haemophilia and parents of children with haemophilia	Quantitative cross-sectional	2014	Haemophilia A Haemophilia B	Parents	Mothers (75.58) Fathers (24.42%)	561	NR	Algeria, Argentina, Canada, China, France Germany, Italy, Spain, UK, US	Being a Carrier of Rare Disease Information Social/Community Support Systems Well-being
Difficulties experienced by caregivers of patients diagnosed with osteogenesis imperfecta (OI): example of a hospital	Mixed-methods	2015	Osteogenesis imperfecta	Parents	Mothers (93.5%) Fathers (6.5%)	46	Mean: 35.52	Turkey	Carer Perceptions Faith Information Practical Quest for Knowledge Social/Community Support Networks Well-being
Through the looking glass: an exploratory study of the lived experiences and unmet needs of families affected by Von Hippel–Lindau disease	Mixed-methods	2015	Von Hippel–Lindau disease	Parents Partners	Mothers (62.5%) Fathers (12.5%) Partners (37.5%)	8	Mean (range): 57 (37–75)	Australia	Information Practical Social/Community Support Systems Well-being
Parenting a child with haemophilia while living in a non-metropolitan area	Qualitative	2015	Haemophilia A Haemophilia B	Parents	Mothers (85.7%) Fathers (14.3%)	7	Range: 25–48	Australia	Acceptance Being a Carrier of Rare Disease Carer Perceptions Information Practical Quest for Knowledge Social/Community Support Systems Well-being
Mucopolysaccharidosis: caregiver quality of life	Quantitative cross-sectional	2015	MPS-I MPS-II MPS-III MPS-IV MPS-VI	Parents	Mothers (100%)	11	Mean (range): 35 (29–43)	Brazil	Practical Social/Community Well-being
A tortuous route to a capable fatherhood: the experience of being a father to a child with severe haemophilia	Qualitative	2015	Haemophilia A	Parents	Fathers (100%)	14	Mean (range): 43.5 (28–57)	Sweden	Acceptance Carer Perceptions Gratitude and Hope Information Practical Social/Community Well-being
Social/economic costs and health-related quality of life of mucopolysaccharidosis patients and their caregivers in Europe	Quantitative cross-sectional	2016	MPS-II (Hunter syndrome) MPS-I-H/S (Hurler–Scheie syndrome) MPS-I-H (Hurler syndrome) MPS- VI (Maroteaux–Lamy syndrome) MPS-IV, subtypes A and B (Morquio syndrome) MPS- III, subtypes A, B, C, and D (Sanfilippo syndrome) MPS-I-S (Scheie syndrome) MPS- VII (Sly syndrome)	Parents Partners	Mothers (98.5%) Partners (1.5%)	66	Mean: 39.6	Italy, Spain, Germany, France, Hungary, Sweden, Bulgaria	Practical
Parents’ experiences of living with, and caring for children, adolescents and young adults with mucopolysaccharidosis (MPS)	Qualitative	2016	MPS-I (Hurler syndrome, Scheie syndrome) MPS-II (Hunter syndrome) MPS-III (Sanfilipo syndrome) MPS-VI (Maroteaux–Lamy syndrome)	Parents	NR	8	NR	Ireland	Acceptance Carer Perceptions Gratitude and Hope Information Practical Social/Community Well-being
Experiences of caregivers of children with inherited metabolic diseases: a qualitative study	Qualitative	2016	Carnitine uptake defect Citrin deficiency Galactosemia Glutaric acidemia type I Glycogen storage disease type 1 Long-chain 3-hydroxyacyl-CoA dehydrogenase deficiency Maple syrup urine disease Medium chain acyl-CoA dehydrogenase deficiency MPS-I Ornithine transcarbamylase deficiency Phenylalanine hydroxylase deficiency Tyrosinemia type I	Parents Grandparents	Mothers (85.7%) Fathers/Grandfathers (14.3%)	21	NR	Canada	Acceptance Carer Perceptions Establish a Routine Information Practical Support Systems Well-being
Difficulties in daily life and associated factors, and QoL of children with inherited metabolic disease and their parents in Japan: a literature review	Literature review	2016	AA CD HM IEM IMD MPS MSUD PA PKU	Parents	Mothers (NR) Fathers (NR)	NA	NA	Japan	Carer Perceptions Information Practical Social/Community Support Systems Well-being
Caregiver burden in haemophilia: results from a single UK centre	Quantitative cross-sectional	2017	Haemophilia A Haemophilia B	Parents	Mothers (80%) Fathers (20%)	20	Mothers, mean: 40.5 Fathers, mean: 42.5	UK	Practical Social/Community Well-being
The burden of bleeds and other clinical determinants on caregivers of children with haemophilia (the BBC Study)	Quantitative cross-sectional	2019	Haemophilia A Haemophilia B	Parents	Mothers (81.35%)	144	Mean (range): 39.8 (24–57)	Germany, Italy, Netherlands, Poland, Sweden, Turkey, UK	Carer Perceptions Disease Severity Practical Well-being
Psychosocial recommendations for the care of children and adults with epidermolysis bullosa and their family: evidence based guidelines	Literature review	2019	DDEB EBS EBS-I JEB KS RDEB RDEB +	Family	NA	NR	NR	NR	Carer Perceptions Information Practical Social/Community Support Systems Well-being
Pathway to diagnosis and burden of illness in mucopolysaccharidosis type VII – a European caregiver survey	Quantitative cross-sectional	2019	MPS-VII	Parents	NR	12	NR	Germany, Spain, Netherlands, Turkey	Carer Perceptions Practical Information Support Systems
Emotional experience in parents of children with Zellweger spectrum disorders: a qualitative study	Qualitative	2019	Clinically similar peroxisome disorder D-bifunctional protein deficiency Zellweger spectrum disorder	Parents	Mothers (67.57%) Fathers (32.43%)	37	Age Category: mean (%) 25–34: 8 (21.6) 35–44: 24 (64.9) 45–54: 3 (8.1) 55–64: 2 (5.4)	US (89.2%) Outside US (10.8%)	Acceptance Carer Perception Gratitude and Hope Information Practical Support Systems Well-being
Assessing the supportive care needs of parents with a child with a bleeding disorder using the Parental Needs Scale for Rare Diseases (PNS-RD): A single-centre pilot study	Quantitative cross-sectional	2019	Factor II deficiency Factor V deficiency Factor VII deficiency Haemophilia A (mild, moderate, severe) Haemophilia B (mild, moderate, severe, factor X deficiency, fibrinogen deficiencies) Bernard–Soulier syndrome Glanzmann’s thrombasthenia Hermansky–Pudlak syndrome Inherited thrombocytopenia Lowes syndrome May–Hegglin syndrome Platelet storage pool defects Platelet release defects Purpura All other platelet defects Thrombotic thrombocytopenia Von Willebrand disease (type 1, type 2, type 3)	Parents	Mothers (56.9%) Fathers (42.8%)	231	Age Category (%): 15–24: 1.3% 25–34: 8.3% 35–44: 55% 45–54: 31% 55 + : 4.4%	NR	Information Practical Well-being
An online survey on burden of illness among families with post-stem cell transplant mucopolysaccharidosis type I children in the United States	Quantitative cross-sectional	2019	MPS-I	Parents	NR	32	NR	US	Practical
Parental health spillover effects of paediatric rare genetic conditions	Quantitative case–control	2020	Brain malformations Epileptic encephalopathies Genetic kidney diseases Mitochondrial diseases	Parents	Mothers (88%) Fathers (12%)	207	Mean: 38.4	Australia	Practical

AA = Argininosuccinic aciduria, CD = Citrin deficiency, DDEB = Dominant dystrophic epidermolysis bullosa, EBS = Epidermolysis bullosa simplex, EBS-DM = Epidermolysis bullosa simplex Dowling–Meara, EBS-K = Epidermolysis bullosa simplex Köbner type, EBS-WC = Epidermolysis bullosa simplex Weber–Cockayne, HM = Hypermethioninemia, IEM = Inborn error of metabolism, IMD = Inherited metabolic disease, JEB-nH = Junctional epidermolysis bullosa non-Herlitz type, KS = Kinder syndrome, MPS = Mucopolysaccharidosis, MPS-I = Mucopolysaccharidosis type I, MPS-I-H/S = Mucopolysaccharidosis type I Hurler–Scheie syndrome, MPS-I-H = Mucopolysaccharidosis type I Hurler syndrome, MPS-I-S = Mucopolysaccharidosis type I Scheie syndrome, MPS-II = Mucopolysaccharidosis type II, MPS-III = Mucopolysaccharidosis type III, MPS-IV = Mucopolysaccharidosis type IV, MPS-VI = Mucopolysaccharidosis type VI, MSUD = Maple syrup urine disease, PA = Propionic acidemia, PKU = Phenylketonuria, RDEB = Recessive dystrophic epidermolysis bullosa, RDEB-mut = Mutilating recessive dystrophic epidermolysis bullosa

### Conceptual model for carer needs/burden

**Fig. 2:**
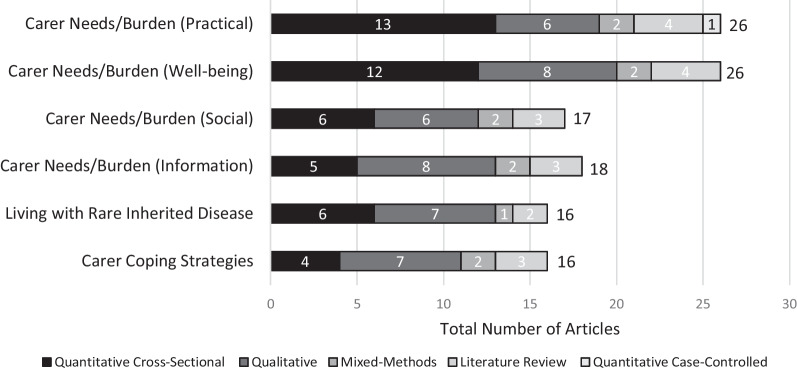
Frequency of themes mentioned across study designs

**Fig. 3:**
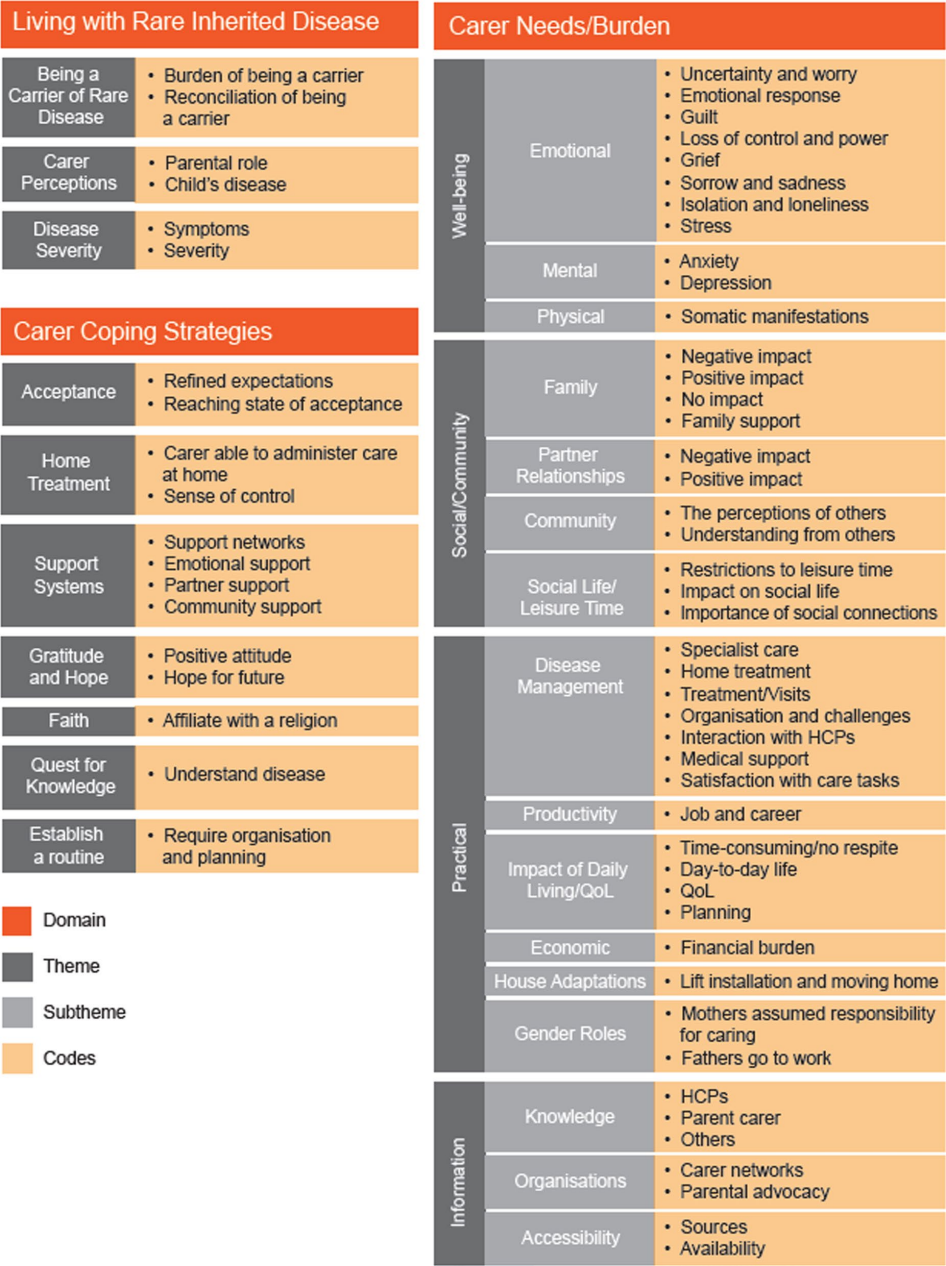
Conceptual model of the needs/burden of carers of people living with rare inherited disease

Key domains/themes and the frequency with which these were mentioned across the included articles are summarised in Fig. 2. The conceptual model (Fig. 3) visualises the three overarching domains that were identified during thematic analysis: (1) Living with Rare Inherited Disease (three themes); (2) Carer Needs/Burden (four themes and 16 subthemes) and (3) Carer Coping Strategies (six themes). The codes grouped under each theme/subtheme are also provided for further illustration.

#### Living with rare inherited disease

The domain of Living with Rare Inherited Disease contains three themes. The theme Being a Carrier of Rare Disease [23–27] describes the burden carers experienced due to their carrier status. For example, parent carers in haemophilia were profoundly burdened by their child’s diagnosis and needed to process haemophilia’s genetic nature [24]. Within this theme, parent carers felt intense guilt for transmitting a RID to their child [23, 24, 26] (defined as ‘transmission guilt’ by Kasparian et al. [27]) and described feeling accused after their child’s birth for not having thought about their carrier status beforehand [23]. The theme Carer Perceptions [23, 24, 26, 28–36] describes the overtly negative attitudes carers held towards their care recipient’s RID, exemplified by carers of people living with osteogenesis imperfecta (OI) describing the disease as ‘a curse’ [30]. Also included are the negative views they held about themselves, such as feeling as though they had failed at being a parent [34] or had lost the parental role they had imagined for themselves [29]. Within the theme Disease Severity [35, 37–39], four studies showed an association between carers’ QoL/burden scores and the severity of their care recipient’s RID, using both carer-specific validated measures (e.g., the Hemophilia Associated Caregiver Burden scale) and generic QoL measures ( e.g., WHOQOL-BREF).

#### Carer coping strategies

Carers adopted a range of different strategies to cope with caring for a person living with a RID, reflected in the domain Carer Coping Strategies. Some were psychological—for example, striving for, and in some cases reaching, Acceptance [24, 26, 29] and refining their expectations of normal life [34]. At other times, carers would adopt positive attitudes and feelings of Gratitude and Hope [29, 34, 36], for instance, carers of children living with haemophilia were thankful for their caring role and responsibility, as they felt these had improved their lives [29]. These carers also hoped that a cure would be developed for haemophilia. [29]

Other strategies were more spiritual whereby carers relied on their Faith [30, 39] to cope. One article outlined how carers of people living with epidermolysis bullosa who did not practise a religion experienced higher burden on certain items of the Family Strain Questionnaire (FSQ) compared with those who did. [39]

More practical coping strategies were also identified, such as when carers would Establish a Routine [33] and the beneficial effect of seeking out and obtaining disease-related information (Quest for Knowledge [23, 24, 30]).

The theme Support Systems [23–25, 27, 30–34, 38, 39] describes the interpersonal support carers received, including that from carer groups [24, 32], employers [23], friends [23], families [23, 25, 27, 30, 32, 33, 39], healthcare professionals (HCPs) [30], schools/day-care facilities [33], psychological services [27], and periods of respite [34]. However, whilst some carers were satisfied with their familial [25] and partner [25] support, others were less so [32, 34, 38] (e.g., carers were displeased with partners’ insufficient knowledge of their child’s treatment [32]). Social support was valued because it helped carers with managing their emotions [34]. However, whilst most coping strategies identified were constructive, some carers employed mal-adaptive strategies, such as withdrawing from others. [30]

Although many carers reported accessing suitable support systems across most diseases, 52.1% of carers of people living with OI reported not receiving any form of social support [30] and highlighted the need for associations/organisation to provide advice [30]. Similarly, carers of people living with inherited metabolic diseases reported needing carer support groups [32]. Patient organisations were poorly advertised in some countries (i.e., Spain and Turkey [28]), and carers identified how interactions with others in support groups were, at times, challenging. [27]

#### Carer needs and burdens

##### Well-being

The domain of Carer Needs and Burdens encompasses the theme Well-being, which details how the Emotional [23–27, 29–37, 39, 39–41, 41–48], Mental [24, 27, 30, 32, 34, 44, 45, 47], and Physical [24, 26, 34, 40, 43, 45, 47, 49, 50] well-being of carers were influenced due to their caring role. The subtheme Emotional Well-being describes carers’ emotional responses towards receiving a diagnosis for their care recipient. Some felt shock [30, 36], anger [36], devastation [36] and, in some cases, relief [24, 26, 34]. Alongside the previously mentioned transmission guilt, additional instances of guilt were felt by parent carers. For instance, fathers of children living with haemophilia felt guilt when going to work and leaving their partners [29]. Carers also felt guilt towards their unaffected children, specifically related to the amount of attention they provided them [23, 29, 34, 44]. Carers often experienced powerful and negative emotions, such as sadness, anger, loneliness and grief [41], as well as feelings of a loss of control [29] and powerlessness [29, 36]. In one case, a carer’s feelings of sadness, anger and guilt negatively affected the relationship between themselves and their child [27], and another article described how, overwhelmed by their situation and grief, carers of children living with haemophilia experienced suicidal thoughts [23]. Many articles referred to the large amounts of stress carers experienced, regardless of the specific RID their care recipient lived with [27, 31, 33, 34, 46, 48]. Certain situations were stressful for carers—for example, carers would become stressed when accidently hurting their child [31]. Furthermore, carers experienced worry about many situations: whether others in their current/future family unit would have the same RID [30, 32], running out of disease management resources (e.g., bandages [44]), and the future of their care recipient. [26, 30, 37, 40]

Within the Mental subtheme of Well-being, anxiety was prevalent in carers [24, 27, 30, 32, 34, 47], including instances of anxious feelings [24, 30, 32, 34], anxiety warranting further clinical assessment (according to the HADS) [27], and Beck Anxiety Inventory (BAI) scores within the cut-off for anxiety [47]. Carers also experienced feelings of depression [30, 45], and two carers’ scoring on the HADS suggested their depression required further clinical assessment. [27]

The Physical subtheme of Well-being outlined the wide range of somatic symptoms carers experienced, including feelings of weakness, shakes, dizziness, headaches, tiredness, ear ringing, weight loss [50], and exhaustion. [24, 26, 34, 45] Whilst it was unclear whether these manifestations were due solely to caring, living with the same condition as their care recipient, or a combination of the two, one article illustrated how carers of people living with haemophilia and who live with a chronic illness themselves reported significantly higher disruption to specific health-related quality of life (HRQoL) domains (Bodily Pain, Physical Functioning and Psychometrically-based Physical Health [40], as measured by the EQ-5D and SF-36), compared with those without a chronic illness. However, another found that carers’ of people living with mucopolysaccharidosis scored only slightly higher (i.e., worse) than average on the physical health domain of the WHOQOL-BREF. [49]

##### Information

Also encompassed by the domain Carer Needs/Burden is the Information theme. Carers’ and HCPs’ disease-related Knowledge [23–25, 27, 29–32, 34, 36, 41, 44] was variable, as was the Accessibility [23, 24, 26–28, 30, 31, 33, 36, 39, 47] of information to carers, and the helpfulness of the Organisations [27, 28, 30–34, 39, 41] surrounding them. Carers of people living with bleeding disorders reported good levels of knowledge about the condition [41], whereas other carers of people living with haemophilia and OI described lacking knowledge [29, 30]. Across a number of diseases, carers felt that HCPs lacked knowledge about their care recipient’s disease [24, 32, 41, 44, 45], and the carers of people living with haemophilia felt ignored/misunderstood by HCPs involved in their child’s care [24]. Regarding healthcare services, some carers even expressed disbelief, anger, and frustration [36]. Carers of people living with bleeding disorders were pleased with HCPs’ knowledge level in a disease-specific centre, but not of those from non-specialist backgrounds [41]. Similarly, carers of people living with haemophilia felt paediatricians and general practitioners lacked knowledge. [24]

Carers relied on several sources to gain disease-specific information, such as the internet [23, 26, 27, 36], social media [26, 33, 36], HCPs [27], and disease-specific society resources [23]. Whilst some carers were satisfied with the general disease-specific information they had access to [27], others expressed a need for more [30, 47] and were dissatisfied with both the quantity and quality of information provided (e.g., concerning available financial and legal support [27]). Two articles described how carers advocated for their child [33, 34], for example at schools and the Government level, due to school’s lack of disease awareness and the need to access specific resources [33].

##### Social/community

Within the theme Social/Community, carers experienced various burdens to their social world, particularly in regard to their Family [24–27, 29–32, 36, 38, 39, 42, 45], Partner Relationships [23–25, 27, 29–32, 36, 40, 44, 45, 49], Community [24–26, 29, 32, 36, 44], and Social Life/Leisure Time [24, 31, 32, 44, 45]. Being a carer often negatively affected their family life and relationships; for example, one article reported that carers of children with epidermolysis bullosa scored poorly on the Family Strain Questionnaire [39]. Another described how fathers were concerned about the relationship they could forge with their child, given the child’s condition, which limited the activities the child could engage in [24]. However, positive influences on families were reported [25, 27], such as other siblings assuming more responsibility within the family unit and furthering their maturity [25]. Moreover, one article concluded that caring for someone living with OI had no significant influence on their social scoring on the WHOQOL-BREF [38]. Relationships between partners were also negatively affected; carers’ role limited the amount of leisure time they were able to spend together [44], and their relationships suffered arguments, deterioration [30], and even separation/divorce [29, 44, 45]. Caring also influenced their decision to not try for more children [29, 45]. However, one article described that carers perceived their marriages as being strengthened by caring for a child living with Von Hippel–Lindau syndrome. [27]

At times, some carers suffered from the negative perceptions of others, experiencing judgement [29], negative reactions when disclosing their child’s disease to others [25], and people staring [36, 44]. Unsurprisingly, some carers felt the need to defend their children from others [36] and wished people understood the nature of caring for someone living with a RID [24]. In terms of the effect on carers’ personal and social lives, they experienced limits on their leisure time [31, 44, 45] and activities [44], as well as their social life. [32, 44]

##### Practical

The theme Practical describes the challenges carers encountered related to Disease Management, [23, 24, 26–29, 29–37, 40, 41, 43–45, 45, 46, 48, 51] impairments to their Productivity [23, 24, 26–28, 33, 37, 38, 40, 43–45, 51], and their Economic [27, 28, 30, 37–41, 43, 45, 48, 52] situation. The Impact of Daily Living/QoL [27, 29, 31, 34, 36, 40, 43–45, 47, 49, 52, 53], Gender Roles [29, 32, 40, 44, 46], and the need for House Adaptations [28, 36] were also encapsulated in this theme.

Disease management practises were often burdensome. For example, carers of people living with epidermolysis bullosa described needing to travel around their home country to attend healthcare appointments [44], and carers living in rural areas of Australia wished to be geographically near a metropolitan centre [24]. Carers of children living with mucopolysaccharidosis spent large amounts of their time within healthcare settings [36], and carers of people living with epidermolysis bullosa [31, 44, 45] and haemophilia [24] found organising care/treatment burdensome. Some carers found accessing disease-specialist care challenging [24] and time-consuming [26], leading carers to recommend the creation of a rare disease centre of excellence to increase clinical practice and understanding [36]. When treatment for their care recipient was available, it was associated with a positive influence on carers (e.g., increased feelings of control [29]), and when carers did receive disease-specialist care, they were generally satisfied with it [33, 41]. However, some interactions between carers, HCPs, and the healthcare system were burdensome, including inadequate collaboration between HCPs and carers, and carers’ uncomfortable emotional reactions to hospitals. [36]

Carers also valued providing at-home treatment for their child’s haemophilia [23, 24, 29], which allowed them to feel capable and regain a sense of control [29]. When carers of children living with mucopolysaccharidosis could receive at-home enzyme replacement therapy for their child, it was a positive experience, with carers describing how it provided structure to their lives. [36]

In addition, carers’ Productivity at work was influenced in many ways by their caring role, including taking time away [33, 51], working fewer hours [23, 28, 51], leaving employment/not working [23, 24, 26, 28, 33, 38, 40, 44], changing to part-time work [40], and experiencing challenges in their work performance [51]. Carers felt unable to follow their own ambitions [44] and said that the diagnosis of their child’s RID disrupted their professional plans [26]. However, some carers saw the event of leaving work as a positive opportunity to further bond with their child. [23]

In terms of Impacts on Daily Living/QoL, some carers felt that their child’s RID minimally affected their lives [27], whilst others perceived their life as a ‘constant battle’ [36] and believed that the RID made short- and long-term planning difficult [31]. Carers of people living with epidermolysis bullosa outlined they were seldom ‘off duty’ [31, 45], and carers of children with Zellweger spectrum disorders reported that their lives were consumed by their role [34]. In one article, 89.2% of carers of people living with sickle cell anaemia reported that they spent 24 h per day caring [43], and in another the mean self-reported hours spent caring for people living with mucopolysaccharidosis was 51.3 h a week [53]. Carers’ HRQoL, QoL, and burden, as measured by both generic and carer-specific measures, were often negatively affected across a range of RIDs. [27, 43, 47, 49, 52, 53]

Many articles described how caring for a person living with a RID was associated with an Economic effect, such as carers having reduced annual incomes compared with matched participants [52], and one article suggesting that a greater level of income in carers of children living with haemophilia is associated with lower burden [37]. Another article reported that scores on specific items of the FSQ (i.e., “difficult to contain anger”, “can’t cope with problems”, “no time for other family members”) differed significantly between carers by family income. However, overall the study found that family burden did not differ by family income [39]. In addition to the economic burden, some carers also needed to adapt their home environments [28, 36], such as installing lifts or moving home [28], to accommodate their child’s safety and behavioural needs. [36]

Furthermore, a number of articles illustrated the Gender Roles involved in caregiving in RIDs. Mothers assumed primary responsibility for the care of their children [29, 32, 40, 44], whilst fathers assumed a breadwinning role [29], working to financially support the family [44] alongside taking responsibility for organisational tasks (e.g., child transportation [44]).

## Discussion

The present targeted literature review aimed to: (1) identify key literature to understand the supportive care needs/burden of informal carers of people living with RIDs, (2) identify the supportive care needs/burdens unique to this group, and (3) develop a conceptual model based on these findings. Through thematic analysis, our conceptual model identified three overarching domains containing 13 themes that describe the needs/burden of these carers.

Our review and that of Pelentsov et al. [12] identified many similar burdens experienced by carers regardless of the inherited nature of the disease. These included a toll on their emotional well-being (e.g., feelings of anger, guilt, loss of control, powerlessness, stress), mental well-being (e.g., feelings of anxiety) and physical well-being (e.g., dizziness, exhaustion, headaches). Carers of patients with RID and more generally rare disease shared numerous challenges: informational (e.g., HCPs’ lack of knowledge), practical (e.g., financial issues) and social (e.g., partner relationships). However, our findings are diversified through the identification of additional evidence of burden attributed to the inherited nature of disease, including reports of clinically relevant anxiety according to validated instruments and qualitative evidence of carer anxiety that the RID may be passed onto any future family member, affecting carers’ decision whether to try for more children. The parental SCNF [12] delineates guilt only in association with carers’ partners and other children; however, our review identified novel instances of transmission guilt, whereby parent carers experienced guilt for passing on a RID to their child [23, 24, 26, 27]. This finding is striking in the context of carers’ scores on the HADS, suggesting clinically relevant depression [47]. Guilt and depression have previously been shown to be associated [54, 55], and guilt is a symptom *of* major depressive disorder [56]. It is therefore possible that the additional burden of transmission guilt contributed to the development of depression/low mood, or that transmission guilt itself is a symptom of carers’ depression/low mood. We suggest this is a hypothesis of interest to be explored by others in future research.

We also identified various coping strategies implemented by carers, some being social or spiritual in nature (Support Systems, Faith), whilst others were psychological (Acceptance, Gratitude and Hope) or practical (Disease Management, Establish a Routine, Quest for Knowledge). This wide range of coping strategies illustrates carers’ resourceful nature; in the face of immense struggles, carers actively sought sources of comfort, relief, and guidance in attempts to mitigate their burden. The theme Faith was a surprising finding, whereby carers in Italy [39] and Turkey [30] used their faith as a coping strategy, as this contrasts with prior work by Speraw et al. [57] (cited by Pelentsov et al. [12]) suggesting the opposite, that carers from the United States of America experienced a crisis of faith following the birth of their child who lives with a disability. This highlights how the use of religion as a coping strategy by carers may differ globally and is possibly influenced by the different religious attitudes towards disability. [58]

Furthermore, our review identified specific positive aspects of caring for a person living with a RID (e.g., siblings gaining maturity). These positive outcomes are not necessarily unique to our carer population but rather align with other theoretical models developed for carers, which posit that developing a sense of self-efficacy and experiencing enrichment in their daily lives as a result of their caring role leads to feelings of accomplishment and a positive perception of caring. [59]

Our findings possess some clinical implications. Firstly, our review identified that carers of people living with a range of RIDs were dissatisfied with the knowledge level of HCPs, specifically non-specialists. Previous research has also shown that HCPs self-report low levels of education and knowledge concerning rare diseases [60]. We therefore wish to echo the sentiments of other researchers [61] in emphasising the need for HCPs to receive formal education regarding rare diseases, including RIDs. Secondly, a previous review has suggested that HCPs’ involvement in and knowledge about providing/offering support to carers differs, with their knowledge level about carers acting as a barrier to support provision [62]. Although our conceptual model is preliminary, we hope it can begin to support HCPs at common points of entry for carers of people living with RIDs to frame these burden/need-related discussions. Moreover, our results highlight that within certain RIDs (e.g., inherited metabolic disease, OI) and in certain countries (Spain, Turkey), there is a need for support groups and patient advocacy organisations.

Our findings also shed light on areas for future research. Whilst our review included informal carers of any type (not only parents), our results were dominated by parent carer samples, particularly mothers. Parents caring for young children also dominated findings, with little focus on caring for adults. Future research should explore the specific experiences of caring for adults living with RIDs, and the experiences of father, sibling and grandparent carers.

Furthermore, Pelentsov et al. [12] argued that many of the parental SCNF domains are interrelated and recommended that future research explore causal pathways between them. We identified quantitative studies suggesting associations between a carer’s QoL and concepts such as child disease severity, income, and religious practice. Such variables are worthy of further exploration in future research as potential mediators or moderators of carer burden. In addition, we noted that the literature included in our review rarely explored the physical burden of carers experienced as a result of living with the same RID as their care recipient. Khair and Mackensen [40] noted that carers who live with a chronic illness (hypothyroidism, hypertension, or being a survivor of acute lymphoblastic leukaemia in childhood) experienced more disruption to their QoL compared with carers who did not. However, none of these carers lived with the same RID as their child. Future research should explore this contributing factor when assessing carer burden and carers’ supportive care needs. Finally, as our conceptual model is preliminary and informed by the literature only, we recommend further ratification through primary research with carers of people living with RIDs.

We advantageously applied a quality appraisal of all articles. However, our review was not conducted to the standards of a systematic literature review, and only two electronic databases were searched; therefore, it is possible that additional eligible articles were absent from our results. Moreover, as 71.9% of the 6000 + rare diseases that exist are genetic [14], our review of 70 diseases may provide only a snapshot of carers’ burden/supportive care needs in RIDs, possibly limiting the generalisability of our results. However, the included disease areas were broad, spanning bleeding, bone, central nervous system, multisystem and inherited metabolic disorders, and we identified many common findings across the included RIDs, suggesting that the results may generalise to many different types of RIDs.

## Conclusions

This targeted literature review bridges the existing evidence gap to conceptualise the burden/needs of carers of people living with a range of RIDs across these areas: social/community, information, physical well-being, emotional well-being, mental well-being and practical needs. We identified further quantitative evidence of clinically relevant depression and anxiety, the unique emotional burden of transmission guilt, worry about future family members having the same RID, and decisions to not have any more children. Our review illustrates the varied coping strategies employed by carers, demonstrating how the existence of a broad range of burdens necessitates an equally broad range of support. Implications for future research include the potential mediators or moderators of carer burden; the experiences of father, sibling, and grandparent carers and carers of adults living with RIDs; and how living with the same RID influences carer’s burden. Although it is subject to further ratification with carers, we hope that our conceptual model can assist HCPs in framing burden/need-related discussions with carers.

## Data Availability

Not applicable. Data sharing is not applicable for this article as no datasets were generated or analysed during the study.

## Abbreviations

HCPs: Healthcare professionals
HRQoL: Health-related quality of life
MPS: Mucopolysaccharidosis
OI: Osteogenesis imperfecta
QoL: Quality of life
RID: Rare inherited disease
SCNF: Supportive care needs framework
UK: United Kingdom
US: United States of America
WHOQOL-BREF: World Health Organization Quality of Life
